# The association between composite dietary antioxidant index and diabetic retinopathy in type 2 diabetic patients: evidence from the NHANES

**DOI:** 10.3389/fnut.2024.1399763

**Published:** 2024-07-16

**Authors:** Shasha Liu, Zhanfang Zhu, Kai Yu, Wei Zhang, Jie Pu, Ying Lv, Zhiguo Tang, Fuqiang Liu, Yongqiang Sun

**Affiliations:** ^1^Department of Cardiology, Shaanxi Provincial People's Hospital, Xi'an, China; ^2^Department of Internal Medicine, Xi'an Jiaotong University Hospital, Xi'an, China; ^3^Department of Cardiology, Pucheng County Hospital, Weinan, China; ^4^Department of Interventional Radiography, Shanxi Provincial People's Hospital, Xi'an, China

**Keywords:** composite dietary antioxidant index, diabetic retinopathy, type 2 diabetes, National Health and Nutrition Examination Survey, antioxidant

## Abstract

**Background:**

Although diabetic retinopathy (DR) is closely related to dietary patterns and oxidative stress, there is little research on the relationship between the compound dietary antioxidant index (CDAI) and DR. This study aims to fill this gap by analyzing data from the National Health and Nutrition Examination Survey (NHANES) to explore the association between CDAI and DR in patients with type 2 diabetes, in order to provide a basis for dietary guidance to prevent DR.

**Methods:**

Data for this study was obtained from NHANES conducted between 1999 and 2020. Information regarding dietary intake was collected through 24 h dietary recall interviews. Multivariate logistic regression analyses and restricted cubic splines (RCS) were employed to explore the association between CDAI and DR. Furthermore, subgroup analyses were conducted to further examine the relationship.

**Results:**

In this study, a total of 2,158 participants were included, with a mean age of 58.87 years. After adjusting for all potential confounding factors, multivariate logistic regression analyses consistently demonstrated a negative correlation between CDAI and DR (OR = 0.94, 95%CI: 0.90–0.98, *p* = 0.007). Specifically, individuals in the highest quartile of CDAI had a significantly reduced risk of DR compared to those in the lowest quartile (OR = 0.51, 95%CI: 0.34–0.75, *p* < 0.001). The RCS analyses further confirmed the linear negative correlation between CDAI and DR (non-linear *p* = 0.101). Additionally, subgroup analyses provided further evidence for the robustness of this association across different subpopulations.

**Conclusion:**

Our study highlights the linear negative correlation between CDAI and DR in type 2 diabetic patients. Further prospective studies are still needed in the future to confirm the role of CDAI in the risk of developing DR.

## Introduction

Diabetic retinopathy (DR) is considered the primary ocular complication of diabetes, affecting approximately 30 to 40% of individuals with diabetes (1). It is a leading cause of blindness among both working-age and older individuals (2, 3). Currently, there are more than 100 million individuals worldwide living with DR, and it is anticipated that the global prevalence and burden of the disease will escalate significantly in the upcoming decades. By the year 2030, it is projected to affect around 130 million individuals, and by 2045, the number is estimated to reach 161 million (4). Extensive research has shown that individuals with DR are at a higher risk of developing various systemic vascular complications, including stroke, coronary heart disease, heart failure, and kidney disease (5, 6). In a longitudinal study from 1,021 patients with type 2 diabetes followed for 9 years, the investigators found an independent association between diabetic retinopathy and a high risk of hypertension (7). Additionally, the economic costs associated with DR and its complications are substantial. In the United States alone, the direct medical expenses related to DR were estimated to be approximately $493 million annually in 2004 (8). Japan also reported medical costs of $1.11 billion related to DR in 2007 (9), although updated data is currently unavailable. Therefore, carrying out research on DR is of great significance, can help prevent and treat this complication, and reduce the pain and economic burden of patients.

The Composite Dietary Antioxidant Index (CDAI), developed by Wright et al. (10). serves as a comprehensive score that evaluates the intake of various dietary antioxidants, including vitamins A, C, E, selenium, zinc, and carotenoids. Selenium, when bound to selenium protein, is known to prevent lipid peroxidation and oxidative cell damage (11). Non-enzymatic antioxidants like vitamins A, C, and E play a vital role in minimizing oxidative changes caused by stress (12, 13). The CDAI was designed based on its combined anti-inflammatory effects on inflammatory markers such as tumor necrosis factor-alpha (TNF-α) and interleukin-1beta (IL-1β). Previous studies have demonstrated that a high CDAI is associated with a reduced risk of various cancers (14, 15), diabetes (16), central obesity (17), as well as decreased all-cause mortality and cardiovascular mortality (18). While oxidative stress is closely linked to DR (19), the relationship between the inflammatory marker CDAI and DR has not yet been fully elucidated.

Therefore, the objective of this study is to investigate the potential association between CDAI and DR among participants in the National Health and Nutrition Examination Survey (NHANES) conducted in the United States. The ultimate aim is to provide dietary guidance to reduce the incidence of DR.

## Methods

### Study population

The study utilized data from NHANES, which is a nationally representative survey conducted by the National Center for Health Statistics (NCHS), a sub-agency of the Centers for Disease Control and Prevention (CDC). The survey targeted non-institutionalized individuals in the United States. Approval for the NHANES protocol was obtained from the NCHS Research Ethics Review Board, and all participants provided written informed consent. The generated and analyzed datasets for this study can be accessed on the NHANES website.^1^

**Figure 1:**
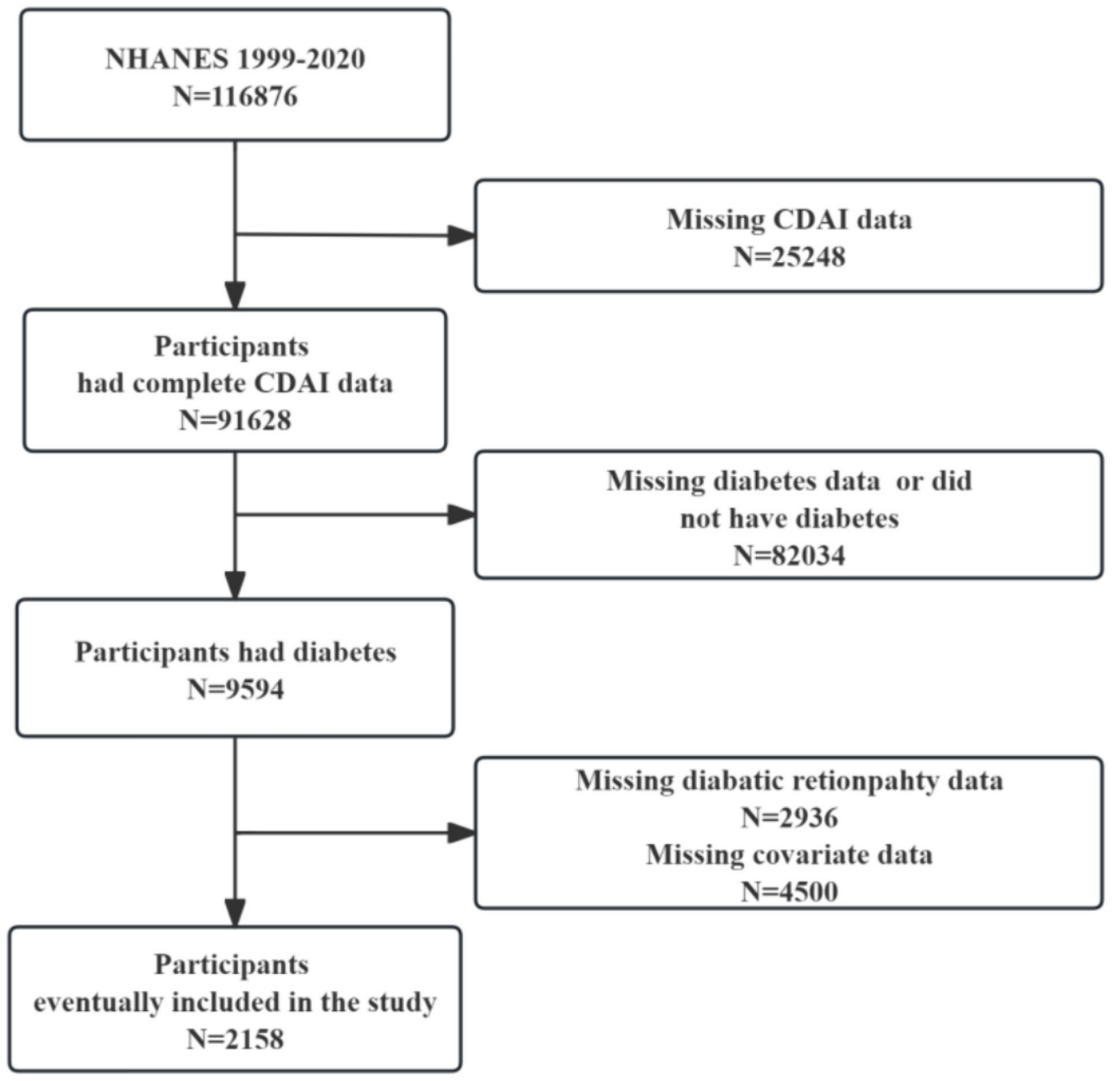
Flow chart of the study subjects.

We downloaded data from 11 cycles of NHANES conducted from 1999 to 2020. Figure 1 illustrates the selection process for the study. Initially, 116,876 participants were surveyed, including 25,248 with missing CDAI data. Participants with a diagnosis of diabetes (*n* = 9,594) were subsequently selected, and ultimately 2,158 study participants were included after excluding those with missing information on DR (*n* = 2,936) and other covariates (*n* = 4,500).

### Primary variables

#### Type 2 diabetes

In this study, type 2 diabetes was diagnosed based on the following criteria: (1) fasting plasma glucose (FPG) ≥ 126 mg/dL; (2) 2 h oral glucose tolerance test (OGTT) ≥ 200 mg/dL; (3) HbA1c ≥ 6.5%; or (4) diagnosed by a physician or the use of antidiabetic medications.

#### DR

DR was defined as those participants who answered “yes” to the question “Has a doctor ever told you that you have DR?”

#### CDAI

Dietary intake data in NHANES were obtained through 24 h dietary recall interviews conducted in mobile examination centers. The interviews were conducted in person and followed up with a second interview over the phone within 3 to 10 days. The types and quantities of foods and beverages, including water, were collected and recorded using the NHANES Computer-Assisted Dietary Interview System. The United States Department of Agriculture’s Food and Nutrient Database for Dietary Studies was used to calculate antioxidant intake, micronutrients, and total energy (20). Nutrient estimates did not include any nutrients obtained from dietary supplements or medications. CDAI was calculated using the method proposed by Wright et al. (10). Standardization was performed for each of the six dietary vitamins and minerals (vitamin A, C, E, selenium, zinc, and carotenoids) by subtracting the population mean and dividing by the standard deviation. The specific calculation method is as follows:


$$\mathrm{CDAI}=\overset{n=6}{\underset{i=1}{\sum }}\frac{\mathrm{Individual}\;\mathrm{Intake}-\mathrm{Mean}}{SD}$$

### Covariates

The covariates included in the analyses were age, sex, race, drinking, smoking, body mass index (BMI), hypertension, alanine aminotransferase (ALT), aspartate aminotransferase (AST), blood Sodium, blood calcium, blood potassium, albumin, total cholesterol (TC), and high-density lipoprotein (HDL). Race was classified as Mexican American, non-Hispanic Black, non-Hispanic White, other Hispanic, and other race. Drinking was categorized as heavy, moderate, mild, former, and never. Smoking was classified as current, former, and never. Hypertension was defined as “yes” or “no” based on the diagnosis criteria of average systolic blood pressure ≥ 140 mmHg or average diastolic blood pressure ≥ 90 mmHg or the use of antihypertensive medications.

### Statistical analyses

All statistical analyses were performed using the weighted methods recommended by the NHANES, and the results presented are weighted. The data was grouped based on CDAI quartiles and the presence of DR. Continuous variables are presented as mean (standard error), while categorical variables are presented as frequency (percentage). Differences between groups were analyzed using one-way ANOVA analyses or *t*-tests for continuous variables and chi-square tests for categorical variables. Three multivariate logistic regression models were conducted to investigate the association between CDAI and DR among individuals with type 2 diabetes: Model 1 (unadjusted), Model 2 (adjusted for age, sex and race), and Model 3 (further adjusted for drinking, smoking, BMI, hypertension, albumin, ALT, AST, sodium, calcium, potassium, TC, and HDL). The results were presented as odds ratios (ORs) with their 95% confidence intervals (95% CIs). Restricted cubic splines (RCS) were used to explore potential nonlinear correlations between CDAI and DR. Subgroup analyses were performed to validate the stability of the results.

All statistical analyses were conducted using R software (version 4.1.3), and a significance level of *p* < 0.05 was considered statistically significant.

## Results

### Baseline characteristics

A total of 2,158 participants, with an average age of 58.87 years (range from 19 to 85 years), were included in this study. Among them, 47.88% were female. The average CDAI of the participants was 0.29. Table 1 displays the clinical characteristics of the participants based on the grouping of DR. Statistically significant differences (*p* < 0.05) were observed in ALT, AST, calcium, albumin, CDAI, vitamin A, vitamin E, zinc, selenium, and drinking between participants with and without DR. Participants with DR had lower values of ALT, AST, calcium, albumin, and CDAI, and a higher proportion of non-drinkers compared to those without DR. Further stratification of clinical characteristics by CDAI quartiles is provided in Table 2. CDAI quartiles showed statistical significance in age, sodium, potassium, albumin, sex, race, drinking, and smoking. The highest CDAI quartile was associated with younger, male, higher sodium, lower albumin, Non-Hispanic White, heavy drinking, and never smoking.

**Table 1:** Characteristics of the study population based on the presence of DR.

	Overall (*N* = 2,158)	No-DR (*N* = 1749)	With-DR (*N* = 409)	*p* value
Age, years	58.87 (0.41)	58.84 (0.46)	59.01 (0.74)	0.843
Sex (%)				0.598
Female	1,005 (47.88)	819 (48.28)	186 (45.91)	
Male	1,153 (52.12)	930 (51.72)	223 (54.09)	
Race (%)				0.168
Mexican American	425 (9.15)	346 (8.96)	79 (10.07)	
Non-Hispanic Black	529 (14.38)	426 (13.83)	103 (17.01)	
Non-Hispanic White	797 (63.26)	661 (64.51)	136 (57.17)	
Other Hispanic	210 (5.71)	168 (5.56)	42 (6.47)	
Other Race	197 (7.51)	148 (7.14)	49 (9.28)	
Drinking (%)				0.005
Former	538 (23.50)	421 (22.50)	117 (28.35)	
Heavy	292 (12.43)	240 (12.84)	52 (10.47)	
Mild	737 (36.46)	616 (38.41)	121 (27.02)	
Moderate	217 (11.21)	183 (11.36)	34 (10.44)	
Never	374 (16.40)	289 (14.89)	85 (23.72)	
Smoking (%)				0.350
Former	704 (33.10)	576 (34.03)	128 (28.61)	
Never	1,091 (49.67)	874 (48.94)	217 (53.19)	
Now	363 (17.23)	299 (17.03)	64 (18.20)	
Hypertension (%)				0.206
No	571 (28.24)	479 (29.07)	92 (24.25)	
Yes	1,587 (71.76)	1,270 (70.93)	317 (75.75)	
BMI, kg/m^2^	32.98 (0.22)	32.96 (0.25)	33.07 (0.42)	0.812
ALT, u/l	26.74 (0.58)	27.26 (0.68)	24.22 (1.07)	0.019
AST, u/l	25.12 (0.43)	25.41 (0.50)	23.76 (0.61)	0.041
Sodium, mmol/l	139.02 (0.12)	139.02 (0.12)	139.05 (0.26)	0.906
Calcium, mmol/l	2.35 (0.00)	2.35 (0.00)	2.33 (0.01)	0.005
Potassium, mmol/l	4.16 (0.02)	4.16 (0.02)	4.20 (0.03)	0.160
Albumin, g/dl	4.10 (0.01)	4.12 (0.01)	4.01 (0.03)	<0.001
TC, mg/dl	181.48 (1.35)	181.67 (1.49)	180.57 (3.36)	0.768
HDL, mmol/l	1.24 (0.01)	1.24 (0.01)	1.24 (0.02)	0.921
CDAI	0.29 (0.13)	0.43 (0.13)	−0.40 (0.21)	<0.001
Vitamin A, mcg	594.92 (14.85)	616.39 (16.02)	490.87 (24.59)	<0.001
Vitamin C, mg	74.08 (3.13)	73.70 (3.26)	75.92 (7.25)	0.768
Vitamin E, mg	7.92 (0.18)	8.111 (0.19)	7.02 (0.40)	0.010
Zinc, mg	10.91 (0.18)	11.144 (0.20)	9.80 (0.37)	<0.001
Selenium, mcg	111.57 (1.87)	113.60 (2.11)	101.70 (3.26)	0.002
Carotenoid, mcg	9179.11 (439.59)	9182.17 (488.94)	9164.30 (874.40)	0.985

BMI, body mass index; ALT, alanine aminotransferase; AST, aspartate transaminase; TC, total serum cholesterol; HDL, high density lipoprotein; CDAI, composite dietary antioxidant index. All values are expressed as a proportion (%) or mean ± standard deviation.

**Table 2:** Characteristics of the study population according to CDAI quartiles.

	Total (*N* = 2,158)	Q1 (*N* = 540)	Q2 (*N* = 539)	Q3 (*N* = 539)	Q4 (*N* = 540)	*p* value
Age, years	58.87 (0.41)	59.47 (0.73)	59.38 (0.77)	59.84 (0.72)	57.10 (0.71)	0.022
Sex (%)						0.026
Female	1,005 (47.88)	273 (55.65)	232 (42.41)	245 (47.86)	255 (46.42)	
Male	1,153 (52.12)	267 (44.35)	307 (57.59)	294 (52.14)	285 (53.58)	
Race (%)						0.022
Mexican American	425 (9.15)	103 (9.51)	104 (8.47)	111 (9.63)	107 (9.05)	
Non-Hispanic Black	529 (14.38)	145 (18.24)	130 (13.51)	138 (14.80)	116 (11.63)	
Non-Hispanic White	797 (63.26)	183 (57.44)	205 (66.91)	189 (60.75)	220 (66.87)	
Other Hispanic	210 (5.71)	63 (7.81)	57 (5.26)	44 (4.05)	46 (5.83)	
Other Race	197 (7.51)	46 (6.99)	43 (5.85)	57 (10.77)	51 (6.62)	
Drinking (%)						0.002
Former	538 (23.50)	160 (27.89)	141 (24.21)	123 (23.10)	114 (19.65)	
Heavy	292 (12.43)	71 (12.22)	67 (12.36)	67 (11.84)	87 (13.18)	
Mild	737 (36.46)	139 (25.71)	192 (35.85)	208 (43.89)	198 (39.41)	
Moderate	217 (11.21)	61 (16.12)	40 (6.23)	58 (8.70)	58 (13.76)	
Never	374 (16.40)	109 (18.06)	99 (21.36)	83 (12.48)	83 (13.99)	
Smoking (%)						0.016
Former	704 (33.10)	174 (31.42)	174 (33.80)	171 (29.25)	185 (37.12)	
Never	1,091 (49.67)	258 (44.03)	273 (48.80)	279 (55.84)	281 (49.77)	
Now	363 (17.23)	108 (24.55)	92 (17.40)	89 (14.90)	74 (13.12)	
Hypertension (%)						0.330
No	571 (28.24)	130 (25.57)	145 (28.57)	139 (26.21)	157 (31.86)	
Yes	1,587 (71.76)	410 (74.43)	394 (71.43)	400 (73.79)	383 (68.14)	
DR (%)						0.006
No	1749 (82.89)	417 (76.06)	443 (82.97)	442 (83.64)	447 (87.74)	
Yes	409 (17.11)	123 (23.94)	96 (17.03)	97 (16.36)	93 (12.26)	
BMI, kg/m^2^	32.98 (0.22)	33.55 (0.53)	32.33 (0.42)	32.80 (0.43)	33.24 (0.41)	0.299
ALT, u/l	26.74 (0.58)	25.14 (1.11)	27.77 (1.69)	25.88 (0.76)	27.87 (0.81)	0.121
AST, u/l	25.12 (0.43)	25.79 (1.34)	24.81 (0.84)	25.08 (0.66)	24.89 (0.66)	0.921
Sodium, mmol/l	139.02 (0.12)	139.17 (0.18)	139.40 (0.21)	138.73 (0.19)	138.82 (0.21)	0.031
Calcium, mmol/l	2.35 (0.00)	2.34 (0.01)	2.36 (0.01)	2.34 (0.01)	2.35 (0.01)	0.099
Potassium, mmol/l	4.16 (0.02)	4.13 (0.02)	4.18 (0.02)	4.22 (0.03)	4.13 (0.02)	0.015
Albumin, g/dl	4.10 (0.01)	4.02 (0.03)	4.13 (0.02)	4.10 (0.02)	4.13 (0.02)	0.003
TC, mg/dl	181.48 (1.35)	183.04 (2.84)	185.62 (2.54)	179.19 (2.77)	178.51 (2.47)	0.194
HDL, mmol/l	1.24 (0.01)	1.24 (0.02)	1.24 (0.02)	1.25 (0.02)	1.24 (0.02)	0.990

BMI, body mass index; ALT, alanine aminotransferase; AST, aspartate transaminase; TC, total serum cholesterol; HDL, high density lipoprotein; DR, diabetic retinopathy.

CDAI: Q1 (−6.65, −2.45), Q2 (−2.45, −0.60), Q3 (−0.60, 1.83), Q4 (1.83, 28.07). All values are expressed as a proportion (%) or mean ± standard deviation.

### Association between CDAI and DR

Table 3 presents the results of the multivariate logistic regression analyses of the association between CDAI and DR. The association between CDAI and DR was significant in model 1 (OR = 0.93, 95%CI: 0.89–0.97), model 2 (OR = 0.93, 95%CI: 0.89–0.97), and model 3 (OR = 0.94, 95%CI: 0.90–0.98). Furthermore, when analyzing CDAI quartiles, participants in quartile 4 had a 49% lower risk of DR compared to those in quartile 1 (OR = 0.51, 95%CI: 0.34–0.75).

**Table 3:** The association between CDAI and DR.

	Model 1		Model 2		Model 3	
	OR (95%CI)	*p*	OR (95%CI)	*p*	OR (95%CI)	*p*
CDAI	0.93 (0.89, 0.97)	<0.001	0.93 (0.89, 0.97)	0.001	0.94 (0.90, 0.98)	0.007
CDAI quartiles						
Q1	Ref		Ref		Ref	
Q2	0.65 (0.40, 1.05)	0.080	0.66 (0.40, 1.08)	0.097	0.68 (0.42, 1.09)	0.110
Q3	0.62 (0.40, 0.98)	0.039	0.62 (0.39, 0.97)	0.039	0.67 (0.42, 1.06)	0.086
Q4	0.44 (0.31, 0.63)	<0.001	0.45 (0.31, 0.65)	<0.001	0.51 (0.34, 0.75)	<0.001
P for trend		<0.001		<0.001		0.002

Model 1: no adjusted.

Model 2: adjusted for age, sex, and race.

Model 3: adjusted for age, sex, race, drinking, smoking, BMI, hypertension, Albumin, ALT, AST, Sodium, Calcium, Potassium, TC, and HDL.

BMI, body mass index; ALT, alanine aminotransferase; AST, aspartate transaminase; TC, total serum cholesterol; HDL, high density lipoprotein; OR, odds ratio; CI, confidence interval.

### Dose-response relationship

**Figure 2:**
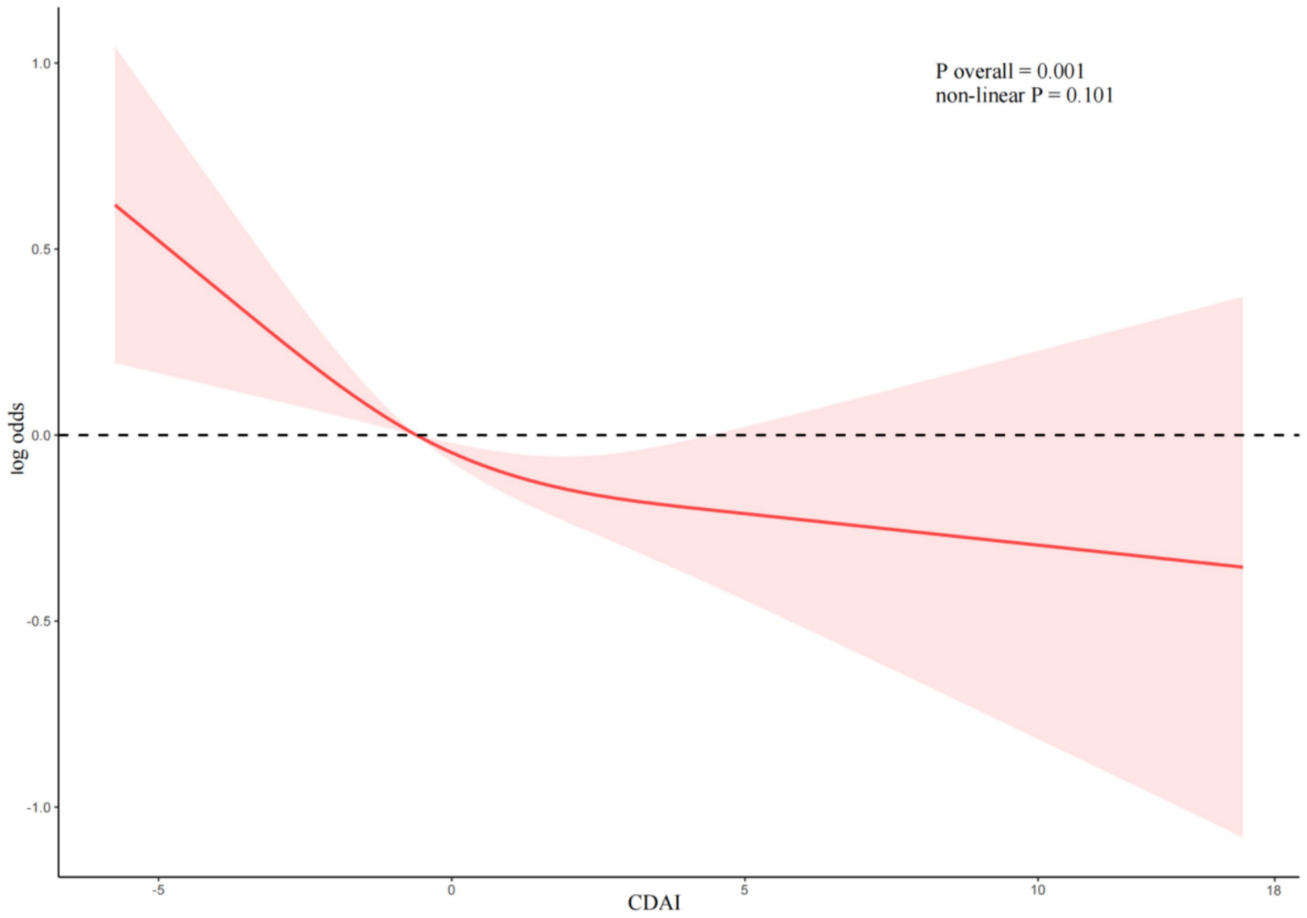
Dose–response relationship between CDAI and DR.

RCS were utilized to analyze the linear association between CDAI and the risk of DR (Figure 2). After adjusting for age, sex, race, drinking, smoking, BMI, hypertension, Albumin, ALT, AST, Sodium, Calcium, Potassium, TC, HDL, the linear association between CDAI and DR remained statistically significant (P overall = 0.001). No non-linear association between CDAI and DR (non-linear *p* = 0.101).

### Subgroup analyses

**Figure 3:**
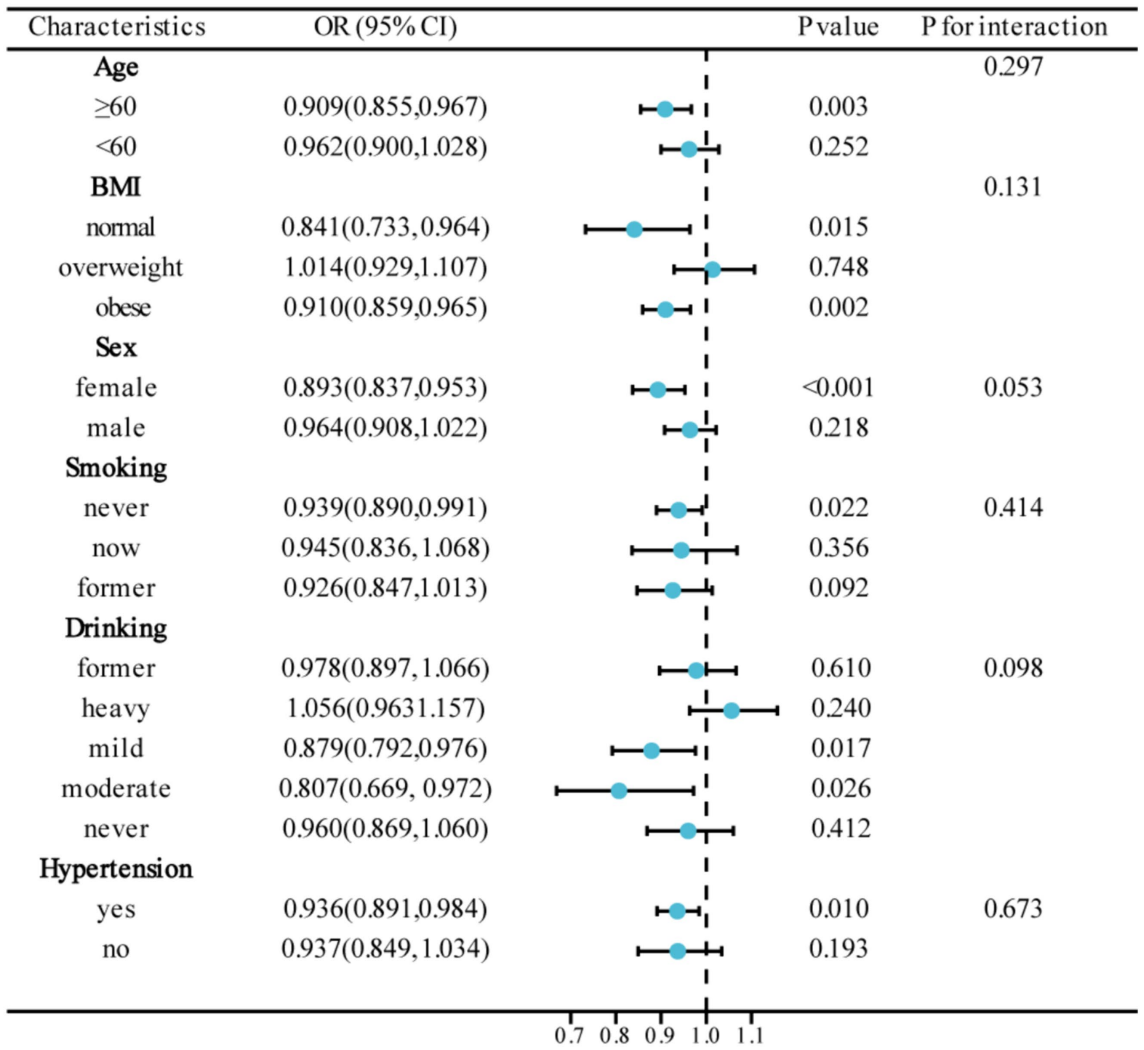
Subgroup analyses of the association between CDAI and DR.

Figure 3 presents the results of subgroup analyses, aiming to study the association between CDAI and DR in different population characteristics. The association between CDAI and DR was found to be significant in subgroups categorized by age, sex, BMI, smoking, drinking, and hypertension (*p* < 0.05). Interaction tests demonstrated no significant influence of different characteristics on the association between CDAI and DR (P for interaction >0.05).

## Discussion

To our knowledge, this is the first study to investigate the relationship between CDAI and the risk of DR within a large sample of individuals with type 2 diabetes. Through adjustment for various confounding factors, we discovered a significant negative association between CDAI and the occurrence of DR among type 2 diabetes patients. Subgroup analyses further substantiated this association between CDAI and DR. These findings highlight the potential role of CDAI in assessing and managing the risk of DR in individuals with type 2 diabetes.

Oxidative stress refers to an imbalance between the production of reactive oxygen species (ROS) and antioxidant defense mechanisms within the body. The accumulation of ROS can lead to the oxidation of various cellular components, including DNA, proteins, carbohydrates, and lipids, ultimately resulting in cellular apoptosis and dysfunction of organs (21). DR, a complication of diabetes, is closely associated with oxidative stress throughout its development (22). Oxidative stress primarily damages cellular mitochondria, leading to retinal cell apoptosis and lipid peroxidation, which in turn contribute to the development of DR (23). Previous studies have demonstrated that inhibiting ROS production can effectively improve DR. For example, a study in diabetic rats showed that treatment with capsaicin reduced ROS levels, improved retinal microvascular permeability, and inhibited DR (23). Another study found that FBXW7, a stabilizer of mitochondrial homeostasis, can reduce ROS generation in hyperglycemic conditions, thereby mitigating the progression of DR (24). Dietary intake of antioxidants can help to protect plasma from ROS and reactive nitrogen species, thereby preventing oxidative stress (15, 17, 25). Dietary modifications present a practical approach to improving the development of DR, with evidence suggesting that consumption of dietary fiber, oily fish, and adherence to a Mediterranean diet can help prevent the onset of DR. Conversely, higher calorie intake has been associated with an increased risk of developing this condition (13, 26). Several clinical studies have investigated the relationship between specific antioxidant micronutrients and DR, finding a negative correlation between the intake of antioxidants such as stavudine, vitamin E (27), flavonoids (28), and DR. These findings collectively highlight the significant role of oxidative stress in the development of DR.

CDAI has been established as a comprehensive measure of dietary antioxidant capacity and has shown associations with the incidence of various diseases. In the context of cardiovascular diseases, higher CDAI levels have been linked to a lower risk of conditions such as hypertension (29), heart failure (30), stroke (31), coronary heart disease (32), and atherosclerotic cardiovascular disease (33). In gynecological diseases, higher CDAI levels have been correlated with a decreased risk of viral infections. A study conducted among healthy women in Italy demonstrated that those with high CDAI had a lower likelihood of positive hrHPV compared to those with low CDAI (34). Moreover, the role of CDAI in individuals with diabetes has attracted considerable attention. Another study utilizing data from the NHANES demonstrated that high CDAI levels not only reduce the risk of developing diabetes but also significantly protect against cardiovascular mortality (35). In studies on diabetic kidney disease, it has been observed that elevated CDAI levels are associated with a lower risk of kidney disease in individuals with diabetes (36). Taken together, these research findings emphasize the significance of CDAI as a marker of dietary antioxidant capacity in various diseases. These findings align with the results of our study, which indicate that higher CDAI levels are linked to a reduced risk of DR Specifically, higher CDAI levels have a protective effect against the development and progression of DR, as well as other diabetic complications.

In our study, we further explored the linear association between CDAI and DR using RCS analyses. The results indicated a negative correlation between CDAI and DR without a clear threshold for CDAI. Subgroup analyses revealed that although there was a directional change in the association among heavy drinkers, the majority of the population still exhibited a negative correlation between CDAI and DR. It is possible that the oxidative stress induced by alcohol consumption may interfere with the protective effect of CDAI.

### Strength and limitation

While this study is the first to investigate the relationship between CDAI and DR in a large-scale population, it does have some limitations. Firstly, due to the cross-sectional study design, we can only establish a correlation between CDAI and DR and cannot prove a causal relationship. Secondly, the use of the 24 h dietary recall method to calculate CDAI may introduce recall bias and may not accurately capture the overall dietary antioxidant capacity. Thirdly, although we considered a number of common confounders in our analyses, other unexplained factors, including renal function and Omega-3 fatty acid intake, may have influenced the results, and caution should be exercised when interpreting study results. In addition, our diagnosis of DR was based on participants’ self-reported physician diagnoses, which may have missed some undiagnosed cases of DR. This reliance on self-report may have led to insufficient sensitivity and specificity of the diagnosis, which could have affected the accuracy of the study. Future studies may consider the use of more objective methods of DR diagnosis, such as fundus photography or evaluation by an ophthalmologist.

## Conclusion

Our cross-sectional study using NHANES data revealed a linear negative association between CDAI and DR in individuals with type 2 diabetes, even after accounting for confounding factors. These findings provide dietary insights for the prevention of DR, and further prospective cohort studies are needed to establish the role of CDAI in DR.

## Data availability statement

The raw data supporting the conclusions of this article will be made available by the authors, without undue reservation.

## Ethics statement

The studies involving humans were approved by National Center for Health Statistics. The studies were conducted in accordance with the local legislation and institutional requirements. The participants provided their written informed consent to participate in this study. Written informed consent was obtained from the individual(s) for the publication of any potentially identifiable images or data included in this article.

## Author contributions

SL: Conceptualization, Data curation, Formal analysis, Investigation, Software, Writing – original draft, Writing – review & editing. ZZ: Investigation, Validation, Writing – review & editing. KY: Validation, Funding acquisition, Writing – review & editing. WZ: Validation, Writing – review & editing. JP: Validation, Writing – review & editing. YL: Validation, Writing – review & editing. ZT: Data curation, Writing – review & editing. FL: Data curation, Writing – review & editing. YS: Conceptualization, Funding acquisition, Methodology, Project administration, Resources, Supervision, Writing – review & editing.

## Footnotes

^1^ https://www.cdc.gov/nchs/nhanes/index.htm
